# Transversalis fascia suture reinforcement may facilitate the performance of electrospun P(LLA-CL) nanoscale fibrinogen mesh in inguinal hernia repair: a prospective single-center cohort study

**DOI:** 10.1038/s41598-023-39391-0

**Published:** 2023-07-26

**Authors:** Kewei Zhang, Leiming Zhu

**Affiliations:** Department of General Surgery, Shanghai Tongren Hospital, JiaoTong University School of Medicine, 1111 Xianxia Road, Shanghai, 200336 China

**Keywords:** Surgery, Medical research

## Abstract

The aim of this study was to evaluate a new electrospun P(LLA-CL) nanoscale fibrinogen mesh performance in real-world clinical practice. A prospective, single-center evaluation of Lichtenstein inguinal hernia repair using electrospun P(LLA-CL) nanoscale fibrinogen mesh in elderly patients with comorbid diseases was conducted between 2020 and 2022. A suture reinforcement of transversalis fascia was applied before mesh implantation. Hernia recurrence, pain score and overall complication rate were measured. A total of 52 inguinal hernias in 48 patients were included. The age of patients ranged from 33 to 95 years, with a median of 78 years. Comorbid conditions included cardiopulmonary disease, organ dysfunction, anticoagulant use, diabetes and smoking. By optimizing the physical condition perioperatively, all patients finished treatment successfully. Four cases recurred secondary to direct hernias or combined hernias and were diagnosed in the first 24 case cohort during follow-up. With surgical procedural modification involving strengthening the posterior inguinal floor by reef-up suturing of the transversalis fascia and the inferior edge of mesh slit to accommodate the spermatic cord, no further recurrence was diagnosed. Postoperative pain was mild and the pain score decreased three months after surgery compared to 1 week after surgery (p = 0.0099). No severe complications occurred, while seroma occurred in six cases. Electrospun P(LLA-CL) nanoscale fibrinogen mesh is safe and effective in repairing inguinal hernias in elderly patients with comorbid disease. A strengthening of the transversalis fascia by suturing may enhance the performance of this mesh.

## Introduction

Inguinal hernia repair is one of the most common surgical procedures performed in the world^1^, with more than 90% repaired with surgical mesh, the mainstream product which dramatically reduces the recurrence rate compared to pure tissue repair^2^. The use of synthetic mesh may induce foreign body reaction and inflammatory response in the host body, culminating in the formation of a scar plate, which brings side effects such as chronic groin pain and stiffness^3,4^. As the mesh implants are permanent, these adverse effects may exist life-long^5,6^.

Biological mesh, which was developed in 1990s avoids the flaws of the synthetic mesh, as they have a different mechanism when implanted into the human body, serving as a regenerative framework that supports matrix remodeling and new collagen deposition, leading to reconstruction of a new mature autologous fascia^7,8^. A few studies has revealed that biological mesh has a comparable recurrence rate and a decreased pain score in the follow-up period compared to synthetic mesh in inguinal hernia repair^9–12^. Most commercial collagen rich mesh is derived from porcine, bovine, or human tissue such as skin, intestinal submucosa and pericardium^7^. Different methods of processing, decellularization and sterilization during manufacture may influence the innate biochemical and biomolecular structure of the collagen scaffold. If the heterogeneous antigenicity is not removed completely, it may critically affect safety and efficacy^7,13^.

A recent multi-center, controlled, single-blind clinical trial showed that a new electrospun nanoscale polylactic acid-ε -caprolactone copolymer (P(LLA-CL)) /fibrinogen(Fg) mesh performed similarly to small intestinal submucosa (SIS) mesh in inguinal hernia repair^14^. This scaffold is prepared by the electrospinning of P (LLA-CL) blended with formulated porcine fibrinogen, which mimicked the extracellular matrix (ECM) environment, with a superhydrophilic property providing good support for cell growth and favorable cell-to-cell and cell-to-matrix interactions without cell debris and genetic material, eliminating the possibility of tissue calcification, immunoreaction and endogenous retrovirus transmission from the tissue sources^15^. However, the trial was conducted in selected people, with a strict exclusion criteria. As inguinal herniation is common in elderly patients, with comorbidities like chronic obstructive pulmonary disease (COPD), diabetes, heart disease, liver or renal dysfunction, this study aimed to assess the performance of this new type of mesh in clinical practice.

## Material and methods

Forty-eight consecutive unselected inguinal hernia patients, 47 male and one female, were recruited in this study from July 2020 to March 2022. The inclusion criteria were adults with physical stages I to III as defined by the American Society of Anesthesiologists (ASA). Excluded patients included those with incarcerated hernia and bowel resection, systemic or local infection, those undergoing radiation, chemotherapy or taking immunosuppressive therapy, any who were pregnant or breastfeeding, allergy to porcine body materials and finally any who were noncompliant. This study was approved by ethics committee of Shanghai Tongren hospital (Approval number:2018-041-02) and the research scheme was conducted in accordance with the standards set out in the Declaration of Helsinki. A detailed description of the clinical trial was provided by physicians, and an informed consent form was provided by each enrolled patient.

All surgeries were performed by a skilled surgeon under general anesthesia or local anesthesia. An inguinal incision was made and the external oblique aponeurosis was opened. The spermatic cord was freed by blunt and sharp dissection from the underlying posterior inguinal canal and any indirect hernial sac was dissected completely or transected and reduced to preperitoneal space at the level of internal ring. When a direct hernia was found, the hernial sac was reduced with a transversalis fascia to transversalis fascia embedded suture at the base of the hernia sac. There were two kinds of methods used to implant a 6 cm × 14 cm electrospun nanoscale P(LLA-CL)/Fg mesh (Remodelling™, Biologic Surgery Graft, Shanghai Pine & Power Biotech Co. Ltd.) in this cohort. In the first 24 cases, one end of the mesh was trimmed into a circular shape that was consistent with the inner angle of the inguinal canal. Prolene 3-0 monofilament suture was used to fix the patch to the anterior rectus sheath of the pubic tubercle, with one side of the mesh sutured continuously to the inguinal ligament from the pubic tubercle. The upper part of the mesh was fixed to the oblique abdominal muscle and rectus sheath, by intermittent suturing to avoid damaging the iliac hypogastric nerve and iliac inguinal nerve. If the nerve distribution affected the placement and fixation of the mesh, that part of the nerve was removed. The mesh was slit laterally and the created tails positioned around the base of the cord structures with the superior tail overlapping the inferior tail. The inferior surfaces of the two tails were sutured together and to the inguinal ligament just lateral to the internal ring forming a cross tail.

**Figure 1:**
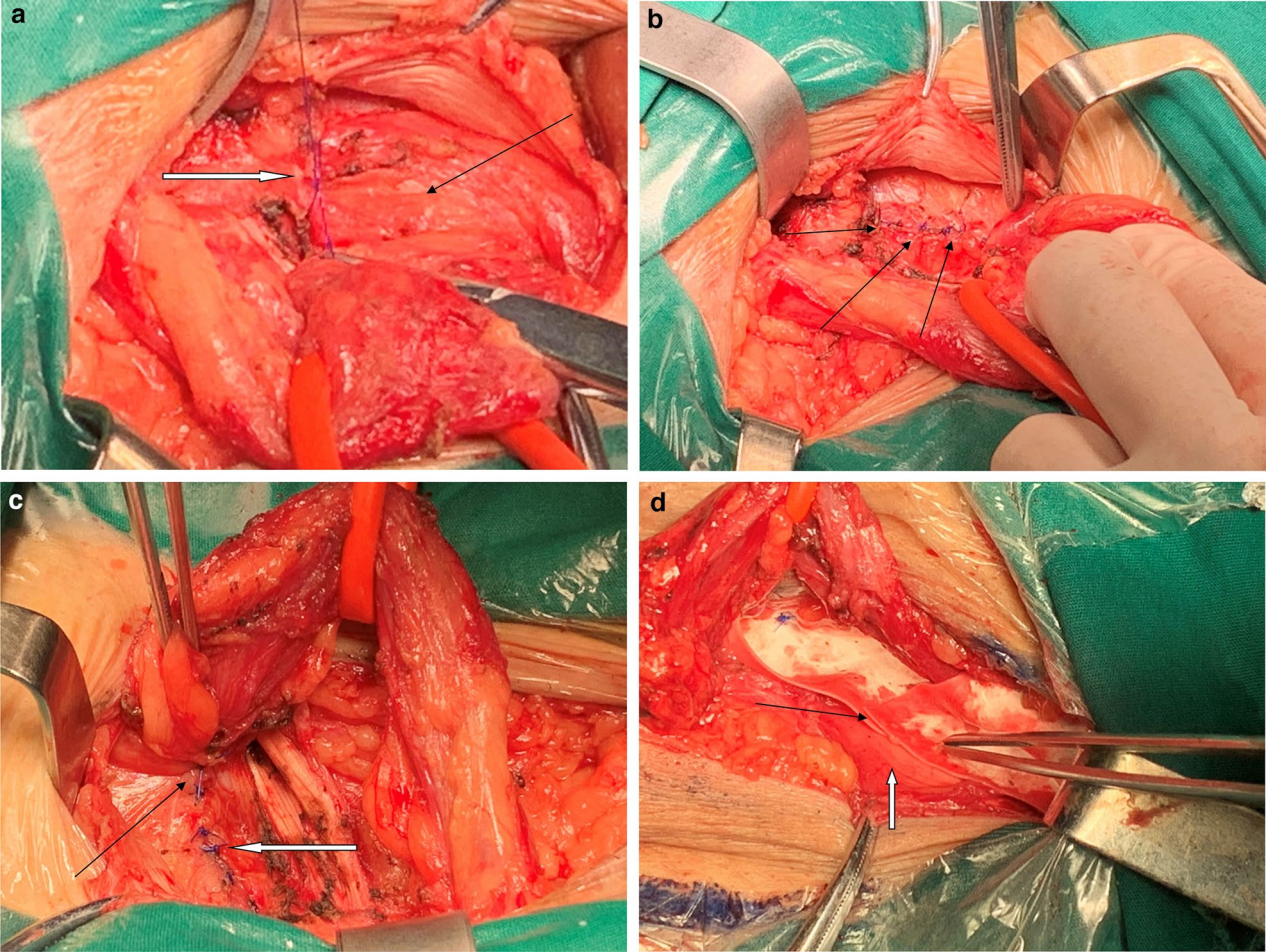
(**a**) Suture with prolene started from pubic tubercle. White arrow: first stitch from pubic tubercle Black arrow: weakened bulging transversalis fascia. (**b**) Transversalis fascia to transversalis fascia suture from pubic tubercle to internal ring. Black arrows: prolene stitches to strengthen the fascia. (**c**) Strengthened and flat posterior floor and reconstructed internal ring. Black arrow: last stitch to define the border of new internal ring. White arrow: stitches of transversalis fascia. (**d**) Implantation of mesh with inferior edge slit to accommodate the spermatic cord. Black arrow: slit at the inferior edge of mesh. White arrow: inguinal ligament.

In the next 24 cases, prior to the placement of mesh, a transversalis fascia to transversalis fascia suture was made with 3-0 Prolene from the pubic tubercle to the internal ring, narrowing the enlarged internal ring and reducing the direct hernial sac, as seen in Fig. 1. The medial part of transversalis fascia was sutured to the shelving edge of the inguinal ligament in patients with poor transversalis fascia on the lateral side to maintain tensile strength after suturing. The mesh was then implanted above the strengthened posterior floor and its tail was slit from its inferior edge to accommodate the spermatic cord. The two slices were overlapped and sutured together to the inguinal ligament and the mesh was fixed by intermittent Prolene suture at the pubic tubercle, inguinal ligament and oblique abdominal muscle. The aponeurosis of the external oblique abdominal muscle was closed using a continuous suture, leaving the spermatic cord in its natural subfascial position and the skin was sutured.

### Follow-up and outcomes

All patients were examined preoperatively and came to the hospital for examination at one week and one and three months postoperatively for follow-up after surgery. Long-term follow-up was conducted by phone.

The primary outcome was defined as the rate of hernia recurrence in the follow-up period. Recurrence was defined by direct clinical examination and /or groin B-ultrasound examination as the presence of the same type of hernia in the same inguinal region that was previously repaired during the follow-up period. Secondary outcomes were chronic pain and postoperative complications such as wound infection, seroma and hematoma. Chronic pain was defined as persistent groin pain or any groin discomfort affecting daily activities that did not disappear by three months after surgery. Pain was evaluated using a visual analog scale (VAS). Complications were assessed during all planned visits and in-between visits when patients came to the hospital.

### Statistical methods

Values for all continuous variables were expressed as means ± standard deviation (SD) and the differences between two groups were examined by the Student’s t test. The classification data were expressed as a percentage and the comparison was performed using the Chi-square test. A *P* < 0.05 was interpreted as significant. Calculations were performed with SPSS 23.0 statistical software (IBM Inc, Armonk, NY).

## Results

Forty-eight patients, including 47 male and one female, gave informed consent to participate in the study, with a total of 52 inguinal hernias repaired. No anesthetic complications or postoperative deaths occurred. Patients base line demographics are shown in Table 1. The patients age median was 78, ranging from 30 to 95. The ASA level ranged from grade I to grade III, encompassing a wide variety of comorbidities before surgery. These comorbid diseases included heart disease, diabetes, COPD, heart, liver or renal dysfunction, or anticoagulant medication and 52.1% of patients had more than one comorbid disease. The physical status of patients was optimized to meet the operation requirement perioperatively. The level of serum glucose were maintained not to exceed 10 mmol/L on the day of surgery. In patients taking anticoagulants, clopidogrel or warfarin were replaced by low molecular heparin for five days before the operation, while aspirin therapy was unaffected except for the day of operation. The cardiac, hepatic, pulmonary and renal function of patients were adjusted according to the opinion of physicians, to ensure physical indices were as suitable as possible for undergoing the operation. Local anesthesia was used in 29 cases (60%) of patients. All patients had completed hernia repairs and early mobilization and eating and drinking were instituted as soon as possible after surgery. The mean follow-up time was 22.9 months, ranging from twelve to 32 months.

**Table 1:** Demographic of the cohort.

Variable	
Age (median; range)	78; 33–95
Male sex (%)	97.9%
BMI kg/m^2^ (mean ± SD)	22.4 ± 2.5
ASA classification, n (%)	
Class I	8 (16.7)
Class II	17 (35.4)
Class III	23 (47.9)
Comorbid conditions	
Cirrhosis with ascites, n (%)	2 (4.2)
COPD/asthma/chronic bronchitis, n (%)	6 (12.5)
Diabetes, n (%)	9 (18.8)
Hypertension, n (%)	25 (52.1)
Coronary artery disease, n (%)	16 (33.3)
Heart dysfunction, n (%)	5 (10.4)
History of percutaneous coronary intervention, n (%)	9 (18.8)
History of cerebral infarction, n (%)	9 (18.8)
Anticoagulant use after cerebral infarction or coronary stent implantation, n (%)	11 (22.9)
Hemiplegia, n (%)	1 (2.1)
Dilated cardiomyopathy, n (%)	1 (2.1)
Parkinson’s disease, n (%)	2 (4.2)
History of pacemaker implantation, n (%)	4 (8.3)
Aortic valve stenosis, n (%)	1 (2.1)
Hyperplasia of prostate, n (%)	8 (16.7)
Renal dysfunction, n (%)	2 (4.2)
Active smoking, n (%)	8 (16.7)
Atrial fibrillation, n (%)	4 (8.3)

The bladder was accidently perforated in one patient where it protruded as part of a large hernial sac. It was repaired and reduced to the preperitoneal space, then the transversalis fascia was sutured and the mesh implanted. A urinary catheter was placed for one week after the operation and the patient recovered without complications. Another two cases of incarcerated hernia were included in this cohort. No intestinal necrosis or perforation were found during surgery and the movement and color of injured intestine was normal. The biological mesh was implanted successfully and the rehabilitation of these patients was uneventful. Operation characteristics are described in Table 2.

**Table 2:** Operation characteristics.

Characteristics	N	
Anesthesia		
General	19	
Local	29	
Hernia type		
Unilateral indirect inguinal hernia	26	
Unilateral direct inguinal hernia	12	
Unilateral combined inguinal hernia	6	
Bilateral inguinal hernia	4	
Incarcerated inguinal hernia, with no bowel resection	2	Included in unilateral indirect inguinal

Four recurrences were found in the first half of this 48-patient cohort, where the transversalis fascia were not reefed up by suturing as part of the procedure. The recurrences were diagnosed 4 to 8 months after operation, secondary to three cases of combined inguinal hernia and one case of direct hernia. Three cases accepted a second inguinal hernia repair operation. Two patients were examined by laparoscopic procedure to explore and repair the recurrent hernia, during which a defect near internal ring area was found and synthetic mesh was used to secure the repair. A Lichtenstein procedure with synthetic mesh was adopted in another patient with a direct hernia recurrence. One patient refused to accept a second repair. The recurrences characteristics were shown in Table 3.

**Table 3:** Characteristics of the recurrences.

Case	Gender	Age (years)	BMI	Original hernia type	Time to recurrence (months)	Recurrent hernia type	Second repair method
1	Male	83	22	Combined	5	Indirect	Laparoscopic repair
2	Male	81	21.9	Combined	8	Direct	Lichtenstein repair
3	Male	68	21.2	Direct	4	Unknown	–
4	Male	77	23.7	Combined	8	Indirect	Laparoscopic repair

After a review of these recurrence cases, the surgical procedure in the second half of the study cohort was modified to include a reef-up suture of the transverse fascia to strengthen and provide a flat plane behind the implanted mesh and a inferior slit of the biological mesh to better surround the root of spermatic cord. Patients with this procedural modification had no recurrence during the follow-up period.

Other complications are shown in Table 4. The organ functions of those patients with comorbid diseases were well protected in the immediate follow-up period. Obvious seroma were found in six cases postoperatively, which in three cases progressed to wound redness and swelling. Purulent fluid was flushed from the wound and extended to the surface of the mesh implant. All cases of infection were treated conservatively with wound drainage and dressing change and recovered without the need for mesh removal.

**Table 4:** Post-operative complications.

Postoperative event	N (%)
Recurrence	4 (8.3)
Seroma	6 (12.5)
Hematoma	0
Scrotal hematoma	0
Wound infection	3 (6.3)
Orchitis	0
Urinary retention	1 (2.1)
Dehiscence	2 (4.2)
Deep vein thrombosis	0
Pulmonary embolism	0
Respiratory insufficiency	0
Pneumonia	0
Worsen liver function	0
Acute renal failure	0
Myocardial infarction	0
Acute heart failure	0
Paralytic ileus	0
Small bowel obstruction	0
Death	0

The VAS score was collected at one week and three months after the operation. The groin pain and groin discomfort were not as severe in this cohort with a VAS score of zero to two points and there was decreased pain score at three months compared to one week postoperatively (p = 0.0099).

## Discussion

Biological meshes have evolved in response to a growing need for safe alternatives to permanent synthetic meshes which have the complications of chronic pain, infection, erosion and fistula formation^5,6,16–18^. Biologic meshes serve as a regenerative framework that attract early cellular and vascular infiltration, support site-specific tissue remodeling and new collagen deposition into the host’s tissue, resulting in the reconstruction of a new and mature autologous fascia^7,8^.

Unlike most popular biological meshes which are extracted from the dermis or submucosa tissues of humans, porcine and bovines, a newly developed electrospun nanoscale P(LLA-CL)/Fg mesh has proved its efficacy in a multicenter, controlled study. This mesh is derived from formulated porcine fibrinogen, which is a plasma protein that plays a pivotal role in wound healing and ECM remodeling, is abundant in supply and is assumed to lower the risk of immunoreactions and tissue calcification^19^. With the use of electrospinning technology, the deposition of fibrinogen amino acid residues onto the scaffold surfaces improves its biocompatibility and hydrophilic properties favoring cell- cell and cell–matrix interactions^15^. However, the clinical trial was conducted in selected patients, whose age ranged from 20 to 75 years, and the exclusion criteria ruled out those with one or more comorbidities^14^. Geriatric patients with comorbid disease represent a significant percentage of inguinal hernia patients, as these comorbidities are inherent risk factors to the formation of hernias and the tissue regeneration in these patients is less than younger individuals.

The performance of this new biological mesh needed to be clarified in clinical practice and our study included a significant percentage of elderly patients, with a maximum age of 95 years and a median age of 78 years. A wide range of comorbid diseases, including cardiopulmonary disease, organ dysfunction, anticoagulant use and diabetes were common in these patients. Two with incarcerated inguinal hernias but without bowel resection and one case of perioperative bladder injury were also included. This cohort to a certain extent reflected the everyday clinical profile of such clients and the result of hernia repair with electrospun nanoscale P(LLA-CL)/Fg mesh was encouraging. All patients underwent the surgical procedure safely and rehabilitated well. No infection happened to incarcerated cases or the case with bladder injury.

There were four cases of recurrence, all in the first half part of this cohort. As this was a prospective study, recurrence was the primary concern, so the study was paused after finishing the first 24 cases. The medical records of all four recurrent cases and the findings in the revision operation were carefully reviewed. The inguinal hernia was repaired according to the classic Lichtenstein procedure in the first half part of cohort. Electrospun nanoscale P(LLA-CL)/Fg mesh is thicker than SIS mesh and less stretchable compared to polypropylene (pp) mesh. In some cases, the overlap of the two tails and suture to the inguinal ligament to build a cross-tail pattern was not as easy as with pp mesh and may stress the tail of the mesh which may have prevented a secure repair. This may have been the cause of recurrence in the cases where a defect was detected at the internal ring during laparoscopic exploration. The mesh slit was adjusted according to the description of Bellows et al.^10^ where the tail was slit from its inferior edge to better accommodate the spermatic cord in subsequent cases. Another two recurrent cases were due to a direct hernia and a combined hernia. The repeat repair for the combined hernia case revealed a direct hernia recurrence. Martin Duce et al. reported the results of a prospective study of the Shouldice hernia repair technique, where after 18 years of follow-up, there were seven recurrences, five of them secondary to a direct hernia^20^. Bochicchio et al. performed inguinal hernia repair with SIS mesh in 50 patients and at the one-year follow-up, three recurrences were diagnosed, all as direct inguinal hernias^21^. A three-year follow-up in patients who underwent the Lichtenstein repair with a synthetic long-term resorbable mesh called TIGR Matrix also recorded an extremely high recurrence rate in cases with direct and combined inguinal hernias^22^. In cases with large direct hernias, the transversalis fascia of posterior wall was extensively damaged, even in the area around the internal ring, resulting in an incomplete or enlarged ring. In cases without an obvious direct hernia defect, the transversalis fascia is often loose and bulging and a simple reduction of the direct hernial sac with a purse string suture is not enough to regain the integrity of the posterior floor. Ravo et al. believed that a weakened transversalis fascia played a role in inguinal hernia formation and placed the biological mesh under the transversalis fascia or included it in the sac of the direct hernia and reduced it to the retroperitoneum, followed by a two-layer suture of Prolene, taking in the transversalis fascia to transversalis fascia from pubis up to the internal ring and from the internal ring to the pubis. This tissue repair with a reinforcement of biological mesh resulted in a satisfying outcome comparable to synthetic mesh in a 10-year follow-up^23^. Our study also adopted an intermittent transversalis fascia to transversalis fascia suture with Prolene from pubis to internal ring before the implantation of the biological mesh. This maneuver reduced the direct hernia sac, tightened the loosely bulging fascia, better defined the internal ring and provided a flatter strengthened floor for placement of the mesh. This step also proximated the healthy fascia, which made the mesh overlapping with more strong tissue to allow better integration and remodeling. If the fascia was suspected to be weak at the lateral side, the inner part fascia or rectus sheath can be sutured with the shelving edge of the inguinal ligament. These technique modifications were used in the second part of this cohort of another 24 cases. There was no recurrence in the follow-up period for this cohort.

Seroma was reported as a common complication after inguinal hernia repair and more prominently in those with biological mesh implants^24^. Liu et al. reported more than 90% patients had seroma in one week after operation with a porcine SIS mesh implant, regardless of whether a Lichtenstein or transabdominal preperitoneal (TAPP) procedure was used and one month after operation, the seroma occurrence rate dropped to 17.3% in the Lichtenstein group, while in TAPP group the seroma rate remained at 96.7%^11^. Seroma can be secondary to patient-related factors, surgical trauma and foreign body reaction to the mesh. Ansaloni et al. suggested that SIS implantation in people elicits a humoral immune response toward the implant and its α-gel component without clinical signs of rejection^25^ and that this immune response may be related to a postoperative seroma. The current electrospun nanoscale P(LLA-CL)/Fg mesh is different from widely used porcine SIS mesh in its material resource and manufacture process, where is no heterogeneous cell element left behind, theoretically resulting in reduced incidence and severity of seroma. However, six of 48 patients in this study developed seroma. The bioactive substances were released during the degradation process of porcine fibrinogen, which recruited cellular components and factors, triggering a series of regeneration programs afterwards and may be the reason for the formation of seroma in certain people.

In line with other biological meshes, the postoperative pain score and the foreign body sensation in the groin area was mild with the use of electrospun nanoscale P(LLA-CL)/Fg and illustrated the superiority of absorbable biological material compared to permanent synthetics, so its presence in potentially contaminated settings, two incarcerated hernias and one bladder injured case in this study did not require late mesh removal.

### Limitations of the study

The main limitations of this study were the single-center design with small sample size and the short follow-up duration. Large randomized controlled trials representing real-world clinical practice with longer follow-up time, even the analysis of the outcome with rapid evolving artificial intelligence techniques in surgery^26^, are needed to further address the performance profile of this electrospun nanoscale P(LLA-CL)/Fg biological mesh in inguinal hernia repair.

## Data Availability

The data that support the findings of this study are available on request from the corresponding author.

## References

[1] Kingsnorth A, LeBlanc K (2003). Hernias: Inguinal and incisional. Lancet.

[2] Simons MP (2009). European hernia society guidelines on the treatment of inguinal hernia in adult patients. Hernia.

[3] Klinge U, Dievernich A, Stegmaier J (2022). Quantitative characterization of macrophage, lymphocyte, and neutrophil subtypes within the foreign body granuloma of human mesh explants by 5-marker multiplex gluorescence microscopy. Front. Med. (Lausanne).

[4] Klosterhalfen B, Klinge U (2013). Retrieval study at 623 human mesh explants made of polypropylene—Impact of mesh class and indication for mesh removal on tissue reaction. J. Biomed. Mater. Res. B. Appl. Biomater..

[5] Bendavid R (2016). A mechanism of mesh-related post-herniorrhaphy neuralgia. Hernia.

[6] Iakovlev V (2018). A pathology of mesh and time: Dysejaculation, sexual pain, and orchialgia resulting from polypropylene mesherosion into the spermatic cord. Ann. Surg..

[7] Novitsky YW, Rosen MJ (2012). The biology of biologics: Basic science and clinical concepts. Plast. Reconstr. Surg..

[8] Novitsky YW (2013). Biology of biological meshes used in hernia repair. Surg. Clin. N. Am..

[9] Köckerling F, Alam NN, Narang SK, Daniels IR, Smart NJ (2015). Biological meshes for inguinal hernia repair—Review of the literature. Front. Surg..

[10] Bellows CF (2014). Early report of a randomized comparative clinical trial of Strattice™ reconstructive tissue matrix to lightweight synthetic mesh in the repair of inguinal hernias. Hernia.

[11] Liu Y, Cao Z, Yang H, Shen Y, Chen J (2020). Porcine small intestinal submucosa mesh to treat inguinal hernia in young adults using laparoscopic inguinal hernia repair: A retrospective controlled study. Surg. Laparosc. Endosc. Percutan. Tech..

[12] Sun L, Chen J, Li J, Shen Y (2020). Randomized and comparative clinical trial of bovine mesh versus polypropylene mesh in the repair of inguinal hernias. Surg. Laparosc. Endosc. Percutan. Tech..

[13] Harth KC, Rosen MJ (2009). Major complications associated with xenograft biologic mesh implantation in abdominal wall reconstruction. Surg. Innov..

[14] Li S (2019). Electrospun P(LLA-CL) nanoscale fibrinogen patch vs porcine small intestine submucosa graft repair of inguinal hernia in adults: A randomized, single-blind, controlled, multicenter, noninferiority trial. J Am. Coll. Surg..

[15] Liu Z, Li S, Su L (2015). Novel superhydrophilic poly(l-lactic acid-co-ε-caprolactone)/fibrinogen electrospun patch for rat abdominal wall reconstruction. J. Biomater. Appl..

[16] Gukas ID, Massouh F (2011). Serious life threatening complication 5 years after laparoscopic totally extraperitoneal hernia repair: Case report and discussion of the literature. Hernia.

[17] Li J, Cheng T (2019). Mesh erosion into urinary bladder, rare condition but important to know. Hernia.

[18] Kunishige T (2013). A defect of the abdominal wall with intestinal fistulas after the repair of incisional hernia using Composix Kugel patch. Int. J. Surg. Case Rep..

[19] Taylor DA, Sampaio LC, Ferdous Z, Gobin AS, Taite LJ (2018). Decellularized matrices in regenerative medicine. Acta Biomater..

[20] Martín DA (2021). Results of Shouldice hernia repair after 18 years of follow-up in all the patients. Hernia.

[21] Bochicchio GV (2014). Biologic vs synthetic inguinal hernia repair: 1-year results of a randomized double-blinded trial. J. Am. Coll. Surg..

[22] Ruiz-Jasbon F, Norrby J, Ivarsson ML, Björck S (2014). Inguinal hernia repair using a synthetic long-term resorbable mesh: Results from a 3-year prospective safety and performance study. Hernia.

[23] Ravo B, Falasco G (2020). Pure tissue inguinal hernia repair with the use of biological mesh: A 10-year follows up. A prospective study. Hernia.

[24] Köckerling F (2018). What is the evidence for the use of biologic or biosynthetic meshes in abdominal wall reconstruction?. Hernia.

[25] Ansaloni L (2007). Immune response tosmall intestinal submucosa (surgisis) implant in humans: Preliminary observations. J. Invest. Surg..

[26] Taher H, Grasso V, Tawfik S, Gumbs A (2022). The challenges of deep learning in artificial intelligence and autonomous actions in surgery: A literature review. Art Int Surg..

